# Putting Your Money Where Your Mouth Is: Why Sustainability Reporting Based on the Triple Bottom Line Can Be Misleading

**DOI:** 10.1371/journal.pone.0119036

**Published:** 2015-03-20

**Authors:** Larissa Shnayder, Frank J. van Rijnsoever, Marko P. Hekkert

**Affiliations:** Innovation Studies, Copernicus Institute of Sustainable Development, Utrecht University, Heidelberglaan 2, 3584 CS, Utrecht, The Netherlands; US Army Engineer Research and Development Center, UNITED STATES

## Abstract

In the packaged food industry, Corporate Social Responsibility (CSR) is an informal requirement for which firms account through sustainability reporting. CSR behaviors are often reported and analyzed using the Triple Bottom Line (3BL) framework, which categorizes them as affecting people, planet, or profit. 3BL is useful in determining which of these categories is most elaborated upon by the firm, but has a limited scope and many documented criticisms. This paper aims to address the aforementioned insufficiencies by augmenting the 3BL framework with two important attributes of CSR practices: (1) the presence of change in core firm behavior of the firm itself or of others in the supply chain, and (2) whether the behavior qualifies as being outside of the firm’s normal business practice or is something that they might have done anyway. We qualitatively analyze CSR behaviors described in sustainability reports and interviews from major players in the packaged food industry and categorize them using these attributes as a supplement to 3BL. This enables us to separate the behaviors from their framing and analyze them more critically. Our results demonstrate how the visible CSR efforts of a firm can be misleading at first glance. Using only 3BL, we find that the CSR focus of firms in this industry is people. We then discover that the codes focusing on people (as opposed to planet or profit) require the least amount of real structural change from a firm or its supply chain partners, and thus arguably, the least amount of effort. We also find that behaviors that focus on planet require the most effort within the firm itself, but for behaviors involving supply chain partners, effort is required for behaviors in all three categories. Finally, we find that CSR behavior that is related to planet tends to go beyond normal business practice.

## Introduction

The term “Corporate Social Responsibility” (CSR) is becoming increasingly well-known in the packaged food industry. Influential firms like Unilever, Heinz and PepsiCo are quickly taking action to reduce packaging material, source sustainably, innovate low-fat/low-salt/low-sugar alternatives, and even sponsor local sports teams. For multinational packaged food companies, CSR seems to be all but a requirement for survival. They write down their efforts in annual sustainability reports, where they meticulously account for their CSR practices to their stakeholders. In these publicly available documents, organizations outline the details of their responsible innovations, product changes, corporate initiatives, community service projects, and new environmentally friendly practices.

Because efforts are taken on an individual basis, each firm implements and accounts for CSR in its own way, which has led to a broad spectrum of activities being lumped together into the CSR category. There is currently no generalized understanding of CSR that untangles the present diversity of these CSR activities, particularly in the packaged food industry.

There are a few commonly accepted ways of categorizing sustainability-focused behaviors. Carroll [1] identifies the components of CSR as economic, legal, ethical, and philanthropic–he calls this the CSR pyramid. Scott Cato [2] advocates a single-pillar approach in which social justice is an (or perhaps *the*) inherent focus. But the most widely used framework is the triple bottom line approach (3BL) [3], which separates behaviors into the categories of people-, planet-, and profit-based. This approach is useful for identifying which aspects of CSR are mostly elaborated upon by the industry. It has, however, been criticized for its subjectivity and its inability to systematically quantify and aggregate the people and planet paradigms. Furthermore, it is seen as limited in its capacity to paint a complete picture of CSR in the packaged food industry or objectively measure social performance, and can even be misleading [4, 5]. We analyze these critiques and identify the insufficiencies that must be given attention for the successful execution of our empirical study. The aim of this paper is to address the aforementioned insufficiencies by augmenting the 3BL framework with two important attributes of CSR practices: (1) the presence of change in core firm behavior of the firm itself or of others in the supply chain, and (2) whether the behavior qualifies as being outside of the firm’s normal business practice or is something that they might have done anyway. Empirically we classify the self-reported CSR behaviors of firms in the packaged food industry in a way that allows us to see whether firms are indeed “putting their money where their mouth is,” or talking about one thing while doing another. Adding the concepts of core firm behavioral change and normal business practice to 3BL can help to evaluate the actions and behaviors of firms on the basis of their commitment to “social responsibility” and to compare individual corporations against one another. In this way, this analysis helps to create a clearer and more honest map of CSR in this industry. It provides packaged food companies insights into opportunities to differentiate themselves from competitors. Moreover, it can allow stakeholders and consumers to better understand firms’ practices.

To achieve this aim, we take a bottom-up approach by qualitatively analyzing sustainability reports and interviews from prominent industry leaders. We do this because efforts to map an industry’s behavior cannot be successful without input from the firms themselves. Only they know the intricacies of their trade, including pressures from stakeholders that cannot easily be seen from the outside and behaviors that may not be discussed in publicly available documentation.

CSR is a heavily studied topic and the idea to include the firm in mapping or defining CSR is recent. In recent years, studies have been done using input from firms of a specific size, industry, or geographical location[6, 7, 8]. We apply similar principles in our study of CSR in the packaged food industry. Because of the importance of this industry to society, as described in the following section, we determine that a study of type is both warranted and necessary.

We find that packaged food industry sustainability reports are very elaborate on topics and behaviors that directly affect their stakeholders–those that fall into the “People” category, according to the triple bottom line. Fewer planet- and profit-based behaviors are discussed, but the behaviors that *are* mentioned in the two latter categories require more core behavioral change on the part of the firm and the supply chain than the people-based behaviors. Furthermore, planet-based behaviors are more likely to go beyond normal business practice. Using our findings as a guide, we present a more comprehensive and standardized method that firms can use to report their CSR practices. This method takes into account core firm behavioral change and normal business practices, thereby rendering firms’ true CSR effort more visible. Moreover, it facilitates a clearer comparison between firms’ CSR practices.

Theoretically, we contribute to the CSR literature by offering a new perspective on CSR in this industry. By taking into account the change in core firm behavior that specific CSR initiatives require and whether or not they are normal business practice, we offer a broader set of criteria by which CSR behaviors can be evaluated. These criteria go beyond just the effects of the behaviors themselves but also consider the attributes of said behaviors–an approach that can be vital for future empirical research into packaged food industry CSR.

In the remainder of this paper, we first briefly delineate and describe the packaged food industry, and demonstrate why it is important to study CSR in this sector. Next, to map CSR and help explain some of the common themes, we discuss the triple bottom line framework [3] and the theory of Stakeholder Management (SM)[9], as well as the concepts of *core behavioral change* and *normal business practice*, as they are used in our analysis. After this, we present our qualitative methods followed by our results, based on 3BL and our two supplementary dimensions. We finish the paper with a discussion and some concluding remarks, in which we feature practical suggestions for implementing our findings.

## The Packaged Food Industry

It is difficult to precisely separate the “packaged food” industry because of the wide range of food items available on the market today. A broad spectrum definition would include any food product that requires processing: convenience foods, chips and cookies, frozen foods, pastas, sauces, oils and spices, and much more. Fresh produce that is packaged for sale is not included, as it remains relatively unprocessed when it reaches the consumer.

In general, we prefer the term “packaged food” over “processed food” for three reasons. The first is that foods are processed to different degrees. For example, something like pasta from an organic brand that contains only three ingredients, none of which are additives, is not processed to nearly the same degree as ranch flavored tortilla chips with thirty four ingredients, most of which are additives. Second, the term “processed” comes with its own set of biases and opinions as it is often used to describe chips and snacks–foods that are notorious for being unhealthy. “Packaged food” is a more neutral term. Third, the term “packaged” describes the wrapper and not the food itself, thereby distancing people from any feelings about the contents of the wrapper.

There is a large margin of error when measuring the global worth of the packaged food industry. Developing countries are home to an astounding number of unofficial food retailers such as kiosks and prepared food stands. Also, because products are often purchased, processed further, and resold, determining the final point of sale is problematic making it easy to double count. Even so, based on Figs. from Euromonitor International, Forbes Magazine estimates the worth of the packaged food industry at almost $1.6 trillion. This is about one third of the total worth of the food and agriculture sector, worth $4.8 trillion and corresponding to 10% of the world’s GDP [10]. This fact alone makes accountability by packaged food companies exceedingly important. Perhaps more crucially, globalization and the worldwide spread of western urbanized lifestyles ensure that the industry will continue to grow in coming years [10].

Beyond its sheer size and potential for growth, the packaged food industry has some unique features that make studying its CSR practices even more compelling. First, everyone must eat. Consequently, as a consumer, it is very difficult to avoid patronizing the packaged food industry. In that sense, packaged food firms can be compared to utility companies–we cannot avoid them without significant and inconvenient lifestyle changes. Second, food is different from other consumer products in that we put it inside of our bodies, directly affecting health, disease risk, and quality of life. Furthermore, the components of packaged food can affect consumer behavior. Foods containing high concentrations of sugar and other refined sweeteners, refined carbohydrates, fat, salt, and caffeine have been shown to cause addiction, which leads to an overconsumption of these items [11]. Consequently, consumers are more likely to suffer from conditions such as obesity, Diabetes Mellitus Type II, and chronic heart disease [12]. For these reasons, ethical issues are at the forefront of discussions related to this industry and packaged food firms must comply with special and unique government and consumer-driven.

Another distinctive characteristic of packaged foods is that–particularly in the case of ultra-processed packaged foods–they have very long supply chains compared to most other industries. The packaged food supply chain consists of approximately eight points or destinations [13]. This may not seem unreasonably large; until we consider (from our earlier example) that a bag of chips could contain thirty-four ingredients, each of which has its own chain. We have now gone from eight supply chain destinations to a conservative estimate of about 256. Chains this large can be problematic, as it is complicated and sometimes impossible to control the ongoing processes in each of the 256 destinations. As a result, it becomes increasingly difficult to guarantee things like healthiness, environmental friendliness, or human rights throughout the supply chain.

Packaged food is thus a large industry consisting of private firms, but with huge public stakes. Consequently, focusing on this industry is a key requirement of this study as we are studying an industry that is invaluable to society, and as such, warrants a study of type.

## Theory

Before turning to our empirical analysis it is important to have a tentative understanding of sustainability reporting, as well as what CSR might encompass and the principles that can explain CSR behavior. A sustainability report is a public report, put together by an organization to provide information to its stakeholders about the organization’s performance in the field of sustainability. Burritt and Schaltegger [14] identify two perspectives on this practice: the critical path and the managerial path. The critical path argues that sustainability reporting actually hinders sustainability [15] and as such, the practice will die out [14]. The managerial path views sustainability reporting as an aid to managerial decision making: an intrinsically valuable tool [14, 16]. Regardless of whether it helps or hinders sustainability in industry, the fact remains that the practice has swiftly swept industries (including packaged food) to become the norm [17] and a window into firms’ CSR-related dealings for stakeholders.

The concept of CSR is broad and has been approached from a variety of angles. Other authors have already reviewed the CSR literature and grouped, organized, or consolidated it. Moir [18] reviews different theories and definitions of CSR from theory and industry practice in an attempt to draw a line between ethics and business. Garriga & Mele [19] consolidated the vast and disparate CSR literature and organized it into four groups based on the work by Parsons [20]: economics, politics, social integration, and ethics.

Literature’s (arguably) broadest perspective on CSR is provided by Campbell [21], who sets only two criteria for socially responsible behavior: (1) Knowingly doing no harm to stakeholders; and (2) Rectifying harm done unknowingly as soon as it is discovered and brought to the attention of the firm. This broad definition fits well with our bottom-up approach, since we accept and use all behaviors reported as CSR by the packed food firm without setting theoretical limitations. A broad definition can be used as a sensitizing concept, which can be further elaborated upon according to our aims after the data is collected.

### 1. Explaining CSR: Stakeholder Management

Next, we discuss some motivations for CSR behaviors. A vital point here is that CSR and sustainability reporting are, for all intents and purposes, optional. Even so, an increasing expectation among stakeholders that these firms will participate in CSR does exist. As such, firms have more of an incentive to change their behavior to comply with these expectations. In the case of sustainability reporting, we see that this practice went from non-existent to widespread in the last decade as more and more packaged food firms adopted it. The Global Sustainability Initiative reports that the number of companies in the food processing sector that have participated in sustainability reporting increased from just three in 1991 to over 60 in 2006 [17]. This is due in part to informal guidelines and certifications that firms can obtain based on their sustainability reporting, such as that of the Global Reporting Initiative. From the rapid increase in reporting, it appears that doing responsible things and obtaining reporting certification has become necessary to gain the acceptance of stakeholders. Gaining the acceptance of stakeholders is a core concept in Stakeholder Management theory–a prominent component of the CSR literature.

The notion of “stakeholders” is defined similarly and fairly consistently throughout the literature, with some refinements made throughout the years. Freeman’s [22] definition serves as the base: “*Groups or individuals who can have effects on, or are affected by, the objectives of an organization”*. Kolk and Pinkse [23] cite Starik [24] to point out that organizations and stakeholders can actually *influence* one another. They note that stakeholders can collaborate and stimulate each other to further assert their influence over organizational behavior. These changes in organizational behavior can influence change in the behavior of other organizations [25].

We speak in section 2 about how the packaged food industry affects consumers in a special way, and according to the aforementioned definition of stakeholders, consumers can also influence the packaged food industry. Furthermore, stakeholders in this industry are far from limited to consumers. They can be internal to the organization or external [26]; direct or indirect [27]. Mitchell, Agle, & Wood [28] propose that stakeholders can be identified using three attributes: power to influence the firm, legitimacy of the stakeholder-firm relationship, and the urgency of the stakeholder’s claim on the firm. Based on this broad framework, we cannot specifically identify each individual stakeholder of each individual firm. We can, however, presume that a firm’s stakeholders come from groups with which it regularly interacts. In the case of the packaged food industry, this includes (but is not limited to) supply chain partners, media, consumers, policy makers, and activists.

The idea that stakeholders can influence behavior is one that jumps out when reading the sustainability reports and websites of the packaged food firms in this study. Because of consumer dependence on these products and the long supply chains involved, it makes sense that a lot of different groups are affected by the behavior of these firms and can thus also have an effect on their behavior.

### 2. Dimensions of CSR: People, Planet, Profit

As mentioned above, the activities that are reported as CSR by firms are diverse. Throughout the management and CSR literature, the categories *people*, *planet*, and *profit* are widely used–a framework known as the triple bottom line. The benefits of CSR are generally seen as falling into some combination of those three categories, as initially defined by sustainability consultancy leader John Elkington [3]. The concept is based on the idea that companies who consider three separate bottom lines–people, planet, and profit–are taking into account the full cost of doing business. From this notion, firms (beginning with the Dutch petroleum company, Shell) began to use these three categories to describe their efforts to be more sustainable and responsible [29].

The idea that people, planet, and profit all contribute to the bottom line is an idea that arose from the Brundtland Report, the culmination of an international summit with the goal of uniting to pursue sustainable development [30]. However, it wasn’t until Elkington’s 1997 book was published that the concept really took off. It has since been generalized to many industries, as demonstrated in a multi-industry study by Raar [31]. As such, it can be an instrumental tool in categorizing the different types of CSR that are present in the packaged food industry as well. *People* is the social category. It includes relationships with stakeholders, ranging from wealthy shareholders all the way to community members in villages, who are affected by the behavior of the firm, such as small-scale farmers, communities around operational facilities, or microloan recipients in the supply chain. Also included in this category are internal stakeholders (such as employees,) and of course, consumers. Consumer health is a particularly important concern within this category. *Planet* is the environmental category. It includes everything that has to do with the effects of firms on the earth. Included in this category are topics like energy savings, sustainable sourcing, transitions to renewable energy, and reducing waste. *Profit* is the financial category. It includes everything that pertains to the financial health of the firm. This can be reducing the costs of production, entering new markets, or anything else that builds up the net worth of the company.

Often, the benefits of CSR affect more than one category. For example, in the context of the packaged food industry, a behavior like maintaining product quality could potentially have two benefits: one affecting consumer satisfaction (*people*) and one affecting reputation (*profit*). Similarly, a behavior like reducing energy use can have environmental effects (*planet*) as well as cost-reducing ones (*profit*). It should be noted that occasionally, a fourth “P” is used–*posterity*. We have chosen to omit this P because it is more of a reason for CSR than a category for its effects. Behaviors that benefit people, the planet, and profit are presumably always done for the future of the organization or of the world: posterity.

We could have chosen other frameworks for discussing CSR behaviors–there is certainly no shortage of them in the literature. Many approaches focus on a circular economy, using materials in a way that eliminates waste or makes it safe to enter the biosphere. Some, like the Cradle to Cradle approach, [32, 33] focus specifically on eliminating or upcycling waste. These are simply too specific for our aim. Others, such as the Blue Economy [34], focus on mimicking nature and using it as a source of innovation and efficiency. This philosophy is, indeed, catching on, but has not yet made its way to the packaged food industry. Perhaps in the future, this approach will be relevant to describe food industry behavior, but currently, sustainability reporting in this industry more closely resembles 3BL. Furthermore, 3BL is commonly used in practical discussions of sustainability and continues to be a go-to framework in a variety of fields, both academic and applied [35, 36].

Even so, 3BL is not perfect. This framework for reporting on and assessing CSR has been criticized, despite its popularity and wide use. Sridhar and Jones [4] identify three shortcomings of 3BL. The first is the reliability of the measurement, referring not to *what* is measured but *how* it is measured. The authors allude specifically to the tendency to report on process rather than outcomes, as well as the often seen attempt to aggregate behaviors without an objective way of doing so. This criticism is not novel (see also Norman & Macdonald [5]), and Pava [37] offers a lucid and practical response with the following quote (pg. 108):

One of the major limitations of the business ethics movement, to date, has been the inability to measure and track social and environmental performance in a meaningful, consistent, and comparable way. But blaming the advocates of triple bottom line reporting for this failure is to blame the only group that has noticed this problem and is trying to remedy it. Rather than criticizing triple bottom line reports for their failure to provide a magical number that aggregates ethical performance, academics should understand the real import of 3BL reporting and try to improve it.

In the following sections, as called on by Pava, we show how we improve on 3BL reporting by taking into account core firm behavioral change and normal business practice.

The second shortcoming identified by Sridhar and Jones is the apparent co-existence of the three bottom lines without a demonstration of interdependence. They maintain that behaviors tend to be lumped in just one of the three categories, when in fact, they may affect more than one bottom line simultaneously, causing them to appear contradictory when they may in fact be complementary. There is precedent in the literature for attending to the interdependence of the bottom lines and even the suggestion that 3BL is becoming more holistic and integrative [38]. We methodologically follow this precedent.

The third criticism suggests that 3BL may not be effective as a compliance mechanism–that is, it may not effectively improve compliance with higher sustainability standards. This may, in fact, be the case. For the purposes of this study, we do not expand our investigation as far as compliance as it is not relevant to our aim.

Another criticism of 3BL, as identified by Tatham, Eisenberg, and Linkov, [36] is that the 3BL definition is too general to allow for comparison between firms or industries. On this point, we agree. However, the generality of the framework is necessary for our study, as it is vital to the bottom-up approach that we have taken. As previously discussed, a broad approach allows us to obtain the specifics of CSR behavior empirically, rather than trying to fit the data to an existing mold.

### 3. Change in Core Firm Behavior

Not all CSR behaviors require the same amount of effort. In this paper we classify CSR effort along two dimensions. The first is whether or not the behavior requires radical change from the firm itself. Organizational theorists have long been aware that some organizations implement superficial changes to increase their legitimacy to the outside world, while their core behavior remains effectively unchanged (see for example Hannan & Freeman [39]). Core firm behavior is defined as any behavior that is related to production or the way that the organization does business. For example, changes to actual products, production processes, or fundamental business practices are all changes in core firm behavior. Sponsoring an athletic team is an example of something that is done parallel to the business of the organization, so it is not considered to require a change in core firm behavior.

As argued before, the packaged food industry is part of a long and complex supply chain. Consequently, some of the CSR behaviors that lead to more responsible end-products need to be implemented outside the boundaries of the individual organization, which means that that firms must manage CSR throughout the supply chain [40, 41]. The idea that the supply chain is a required consideration for measuring enterprise sustainability is well explained by Searcy [42].

Each organization along the supply chain can also choose to change its core business practices when implementing a CSR behavior, and in line with SM, they can be pressured to do so by stakeholders, like the packaged food industry firms themselves.

### 4. Normal Business Practice

According to the Behavioral Theory of the Firm [26, 43] organizations dedicate resources, both within and beyond their core activities, to short-term survival. We call these behaviors normal business practices. A behavior is considered normal business practice if a firm would have done it regardless of its CSR agenda. Organizations can also tactically invest their slack resources into activities that lead to strategic change. CSR behaviors that go *beyond* normal business practice can be viewed as such an activity [44, 45]. For example, firms have to maintain a certain level of quality to keep consumers buying their products, making this behavior normal business practice because they would do it regardless of CSR goals in order to manage their relationship with consumers and survive in the short-term. Similarly, innovation is something that is necessary in this sector to stay afloat, as evidenced by the constant stream of new products that rival those of competitors and keep up with the changing preferences of consumers–stakeholders that motivate the firm to exhibit innovative behavior. Conversely, transitioning to renewable energy is an additional project not required to stay in business in the short term, and thus not normal business practice.

## Methods

### Ethics statement

Upon invitation, participants were informed of the goals of the study and explained in detail that their responses would remain anonymous, and what that means. All participants were made aware that the results of the study would be submitted for publication and all gave their consent via phone or email by agreeing to participate. If consent was given by phone, it was noted at the time by the first author. If consent was given by email, there is written record of it. At the start of each interview, participants were again explained about anonymity and the intent to publish. They were also asked for permission to audio record, and were recorded giving their verbal consent. No IRB or ethics committee approval was obtained because it is simply not done in the Netherlands for studies of this type. Such approval is only required when working with vulnerable populations or if participants are actively manipulated in a way that could be detrimental to their physical or psychological health.

For this study, we take a qualitative approach using a constant comparative method as described by Glaser and Strauss [46]. We begin with a meaningful classification of the data–the triple bottom line approach. This method of classification is well established in the literature and has become a gold standard for evaluating CSR. We then code the data and identify new relationships and classifications, culminating in our two added dimensions. Next, we examine how the triple bottom line and these two dimensions are related. This leads to new theoretical insights into the assessment of CSR in the packaged food industry.

### 1. The Data

We analyzed qualitative data from two sources–publicly available sustainability reports from the organizations in question, and transcripts of interviews with middle-level managers who work in specialized CSR departments or departments that are actively involved in CSR, executed by the first author of the present article. To obtain a good overview of the industry, we chose a mix of medium and large multinational firms, including well-known prominent industry leaders. We did limit ourselves to multinational organizations, as the packaged food industry is internationally oriented [47] and our focus is on the industry as a whole.

The sustainability reports came from sixteen organizations and consisted of a total of 992 pages. Firms were chosen based on four inclusion criteria: (1) The firm has packaged food brands; (2) The packaged food brands have products that meet our definition of packaged food (see Section 2); (3) The firm has an office in the Netherlands (for interviewing convenience); and (4) Sustainability reports are available in English.

In addition to the sustainability reports, we augment the data (for triangulation) with seven semi-structured interviews, all with middle-level managers from firms that also had a sustainability report in our study. The interviews ranged from 25 minutes in length to as long as (in once case) one and a half hours. The interview schedule that was used can be found in S1 Appendix. To recruit participants, letters requesting an interview were sent to one or more managers in each of these firms. Follow-up recruitment phone calls were also made, during which potential participants were informed of the goals of our study, and assured that anonymity would be maintained. Unfortunately, we were unable to achieve the ideal scenario of an interview response from each of the fifteen firms whose reports we analyzed, since not all of them were willing to cooperate with our study. This was communicated through either non-response or a claim of general business. Interviews were conducted via phone, video calling, or in person at the interviewee’s place of employment. With permission from the participants, all interviews were recorded and then transcribed by a third-party agency. The seven completed interviews were vital to answering questions that did not appear in the sustainability reports, giving more candid responses to some of the questions that do already come up in the reports, and touching on some aspects of organizational behavior that go beyond what the organizations make publicly available.

### 2. The Coding

The reports were differently structured between organizations, so it was impossible to limit coding to a particular subject or section. As a result, the reports were coded line by line by the first author with help from a trained and supervised assistant, until theoretical saturation was reached. Atlas.ti was the software used for coding and analysis. For each individual segment (sentence or sentence fragment) of the sustainability reports and the interviewee responses, we identified whether or not it was relevant to our aim. To accomplish this, we assessed whether or not it mentions what CSR means to the firm or specific CSR behaviors of the firm (current or planned for the future). If so, it was coded based on individual terms and topics that arose in the segment. This two-step coding process helped us to determine what aspects of CSR are actual behaviors of the firm, versus actions mentioned in a different capacity (for example, unmet goals). All of the codes with which we work represent the firms’ self-reported perspectives on responsibility, as well as the actions that they claim to take towards said responsibility. We would have preferred to separate *what* CSR is and *how* it is achieved. However, it is not always unequivocally clear whether a segment is answering what or how. For example, take the statement, “The core of our responsibility is our commitment to transparency.” On one hand, the text links “responsibility” with “transparency” implying that transparency is *what* the firm deems responsible. On the other hand, the firm is alluding to its own CSR agenda, in which it is transparent in order to be responsible. This implies a *how* relationship–How is the firm responsible? Therefore we choose a broad criterion that encompasses both *what* and *how*.

In line with our bottom-up approach, we limit ourselves to what the firms deliberately state in their sustainability reports and interviews, and do not attempt to classify on our own whether or not behaviors are actually CSR. The sheer length and diversity of topics in each report lead to a code list of 130, some of which were later merged to higher order concepts. After coding each individual segment of seven of the sixteen sustainability reports, new codes were no longer emerging, which implies that we were approaching theoretical saturation. Rather than continuing to code reports from cover to cover, the remaining reports were read closely to ensure that no new codes would emerge. Any questionable or potentially new areas were coded, while the repetitive content was left alone. The interviews took comparatively disparate directions, and as such, were all coded in their entirety.

### 3. The Analysis

During the coding process it became apparent that some of the text contained quite a few non-specific terms, particularly in the sustainability reports. Though specific activities are sometimes discussed, general or subjective terms such as “harm” and “good” are exceedingly popular, making it sometimes difficult to determine the concrete goals and actions associated with them. As a result, we first distinguish between codes that reflect general statements about CSR goals or actions and concrete statements linked to specific behaviors.

Next, we interpret the codes according to the triple bottom line framework categories: People, Planet, and Profit. We then categorized the concrete codes according to the effort required to accomplish the initiatives, both for the firm itself and along the supply chain. The codes are classified into quadrants based on these two dimensions by considering information from the CSR reports, the interviews, and tertiary information about the industry. Effort is defined in Section 3.3 as a presence of change in core firm behavior. Finally, we determine whether or not the concrete codes went beyond normal business practice. This is a dichotomous choice. All classifications are based on whether or not a behavior is necessary for the short-term survival of the firm; a determination made using a combination of theory and practice based arguments. If a behavior is vital to a firm’s short-term survival (including mandated behavior) then it is classified as being a part of normal business practice. Otherwise, we argue it goes beyond normal business practice. S3 Appendix provides detailed information on specific code classification.

## Results

To give better insight into the data, we first present the uncategorized codes. Table 1 includes a list of all codes linked to general statements that focus on *what* the firm sees as CSR and *how* it engages, as well as a brief description of their content. For each code we explain why we consider them general.

**Table 1:** General codes explained.

CODE	DESCRIPTION
**Be a good corporate citizen**	Includes any behaviors that help a firm to integrate positively into the community, like other citizens. This can mean different things to different firms and different communities.
**Be more efficient**	Includes any behaviors that allow the organization to produce a higher output with lower input. Can include many existing codes but is sometimes used in the sustainability reports without an explanation.
**Be sustainable**	Includes anything that helps to maintain the longevity of the firm, the earth, or humanity. Can include any code that falls into the triple bottom line framework.
**Do no harm**	Widely subjective–unclear *what* or *whom* the goal is not to harm. Can include any codes that represent activities that are not harmful or promote doing no harm to (1) People; (2) The environment (Planet); (3) The firm (Profit); (4) Any other entity that the firm deems important.
**Educate**	Provide education about health, safety, products, processes, behavior, or other subjects to stakeholders or outside communities. Can include a variety of topics and a variety of education recipients.
**Improve society**	Do something positive for society as a whole. Represents an activity, inside or outside the supply chain that does something positive for society as a whole. Can include many of the specific codes on this list.
**Manage stakeholders**	Create and nurture positive relationships with stakeholders. Can include any codes, depending on the needs of stakeholders.
**Plan for the future**	Consider the future of the firm, the planet, or stakeholders when making decisions. It is unclear for *whose* future the firm is planning. Actual behaviors involved in planning can include any codes from the profit category, any codes from the planet category, and many codes from the people category of the triple bottom line framework.
**Process food responsibly**	Keep the processing of food responsible in terms of health, safety, environment, or human rights. Can include any codes that represent activities that are applicable to the production of processed foods. This includes those that have to do with efficiency, food quality and nutrition, waste, and cost.

General codes (Table 1) are codes linked to general statements that focus on *what* the firm sees as CSR and *how* it engages in CSR behaviors. To illustrate the versatility of these codes, here are two examples of quotations coded as “Plan for the future”. The first is in reference to goals that the organization in question has set for 2015:

*“We will include energy per portion on the front of pack plus eight key nutrients and % guideline daily amounts (gda) for five nutrients on the back of pack.”*

This quotation is coded (among other things) as “Plan for the future” because it talks about behaviors that the organization *intends* to engage in. Though it is about concrete behaviors related to transparency, energy, and nutrition, its allusion to future goals means that it should *also* be coded to reflect that. Below is another quotation that is tagged with this code:

*“By 2020, we will help more than a billion people to improve their hygiene habits and we will bring safe drinking water to 500 million people.”*

This quotation covers completely different topics–promoting health/hygiene and access to clean drinking water–yet the reference to a 2020 goal still requires it to be coded as “Plan for the future.” The topics covered in these two quotations are vastly different, yet they qualify to share a code. This is what makes the code general.

Table 2 presents the concrete codes and a brief description of why they are concrete.

**Table 2:** Concrete Codes.

CODE	DESCRIPTION
**Be reliable**	Meet the expectations of stakeholders by remaining consistent
**Be transparent**	Be open about the behavior or status of the firm with stakeholders through reports, websites, or media
**Broaden scope**	Diversify product lines and enter new markets
**Champion fairness/equity externally**	Includes any behavior that contributes to fair wages, humane treatment of people, access to and availability of certain products in underprivileged communities, or anything else that the firm deems fair or equitable outside of its supply chain
**Champion supply chain fairness/equity**	Includes any behavior that contributes to fair wages and humane treatment of employees, or anything else that the firm deems fair or equitable within its supply chain
**Comply with industry standards**	Fulfill expectations set as a standard for industry behavior
**Comply with regulations**	Submit to global, national, or local legislation
**Cooperate**	Work together with other firms or organizations outside of the supply chain
**Do research**	Perform scientific research related to products or processes that affect the firm
**Get feedback**	Receive feedback about the firm and its actions from stakeholders
**Grow**	Boost revenue through increased sales or increase the bottom line or profitability by minimizing costs
**Improve access to clean drinking water**	Help communities with poor access to clean drinking water improve their access
**Improve consumer safety**	Improving safety of ingredients, packaging, and the effects of process on product
**Improve employee safety**	Improve working conditions and safety in production processes
**Improve employee well-being**	Contribute to the health or well-being of employees
**Improve transport efficiency**	Shorten transport distances, switch to lower-emission forms of transport, increase the size of transport loads or other behaviors that reduce the emissions per unit transported
**Increase revenues**	Increase the firm’s income per quarter
**Increase yield**	Produce more with fewer resources
**Innovate**	Develop new products that taste better, cost less to produce, are healthier, or are otherwise improved in some way
**Maintain financial stability**	Maintain the ability to repay debts and continue production without financial repercussions
**Maintain quality**	Keep the quality of products at or above the expectations of consumers
**Manage cost**	Keep costs manageable for consumers or the firm
**Manage nutrition**	Ensure that the firm is producing foods with good nutritional content (not necessarily in lieu of foods with poor nutritional content)
**Manage the supply chain**	Put pressure on the supply chain to participate in certain CSR behaviors. This code alludes specifically toward the firm’s behavior towards others in the supply chain, not the resulting behaviors of others in the supply chain.
**Produce more organics**	Increase number of organic items produced
**Promote good health**	Engage in community-based efforts to encourage healthier lifestyles
**Promote good self-esteem**	Show stakeholders that good self-esteem among consumers or employees is a priority through campaigns, education, or communication
**Protect resources**	Take care not to overuse scarce or valuable natural resources
**Reduce energy use**	Use less energy
**Reduce packaging material**	Use less packaging material
**Reduce waste**	Change production processes to produce less waste
**Reduce water use**	Change production processes to require less water
**Reduce/reuse/recycle**	Change production processes to reduce input, reuse parts that would otherwise become waste, and recycle recyclable materials
**Reduce emissions**	Lower emissions through renewable energy transitions, carbon capture and storage, energy efficiency, or other measures.
**Sponsor events**	Host or contribute to events that promote behaviors or activities within the triple bottom line framework
**Stimulate responsible consumer behavior**	Influence consumers to use the product in a specific way (for example, taking shorter showers when using their shampoo)
**Support human rights**	Contribute to activities that support human rights such as campaigns to limit work hours or NGO efforts to improve work environment
**Support small-scale business**	Contribute to or purchase from small-scale businesses
**Transition to clean refrigeration**	Switch to using environmentally friendly refrigerants or lower energy refrigeration systems
**Transition to renewable energy**	Switch, to some extent, from fossil fuel energy to renewable energy
**Use land responsibly**	Purchase from farmers who consider the impacts of the way they use their land

To illustrate how these codes must be able to be linked to specific behaviors, here is an example of a quotation that is tagged with a concrete code:

*“…we have begun a two-year project addressing malnutrition in Madhya Pradesh, a state with one of India’s highest concentrations of hunger and malnutrition.”*

This quotation carries the code “Manage nutrition”. Though this term is linked to an unelaborated upon project in the quotation, concrete behaviors are required to “address malnutrition.” As such, this is a concrete code. Another more direct example comes from the following quotation:

*“Quantitative studies in Vietnam show we are making some progress, with an increasing number of people cutting down their water consumption.”*

This quotation is linked to two specific behaviors. The first is “Stimulate responsible consumer behavior,” as the research concerns an intervention that does exactly that. The second is to “Reduce water use” in the life cycle of their products.

### 1. Triple Bottom Line

**Fig 1:**
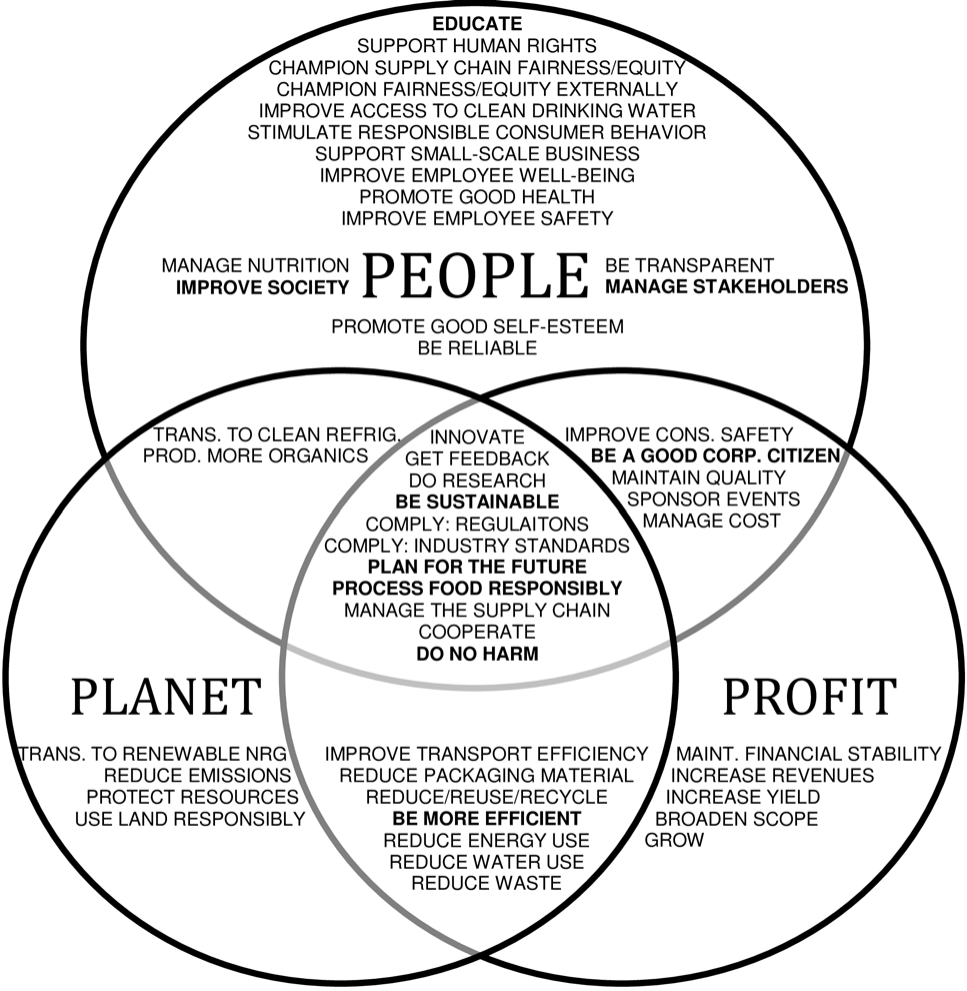
Triple Bottom Line Framework for Packaged Food Industry CSR Behavior.

We use a Venn diagram to show whether each code falls into one or more of the three categories–People, Planet, and Profit (see Fig. 1). We thereby follow the approach by Lozano and Huisingh [38]. A Venn diagram is a visual aid that allows us to see how certain concepts; in this case, codes; fall into one or more of up to three different categories. Such a picture of the categorical distribution allows us to see trends and clusters that might otherwise be difficult to detect.

In this depiction, we have chosen to include the nine general codes (Fig. 1, in bold). They were left in because while they are not necessarily linked to a specific behavior, they do represent a CSR topic or concept that the firm identifies as valuable. From this diagram, we can see that the “People” circle contains the most codes (34), showing us that this is the topic most elaborated upon in the sustainability reports and interviews. “Profit” is the next most elaborated upon (30), and “Planet” the least (25). This means that in the interviews and CSR reports, there are more behaviors or CSR topics mentioned that affect people, as compared to the other categories.

This calls into question the subject of stakeholder management. Is “People” most elaborated upon because it is truly the main focus of the firms? Or do people-focused topics get more attention in sustainability reports simply because they are the most relevant for stakeholders to read about? It is important to note that even though more people-related topics are mentioned, this does not necessarily mean that more money or effort is put into people-related CSR practices. To further investigate this, we must look at the data through our two additional lenses.

### 2. Change in Core Firm Behavior

**Fig 2:**
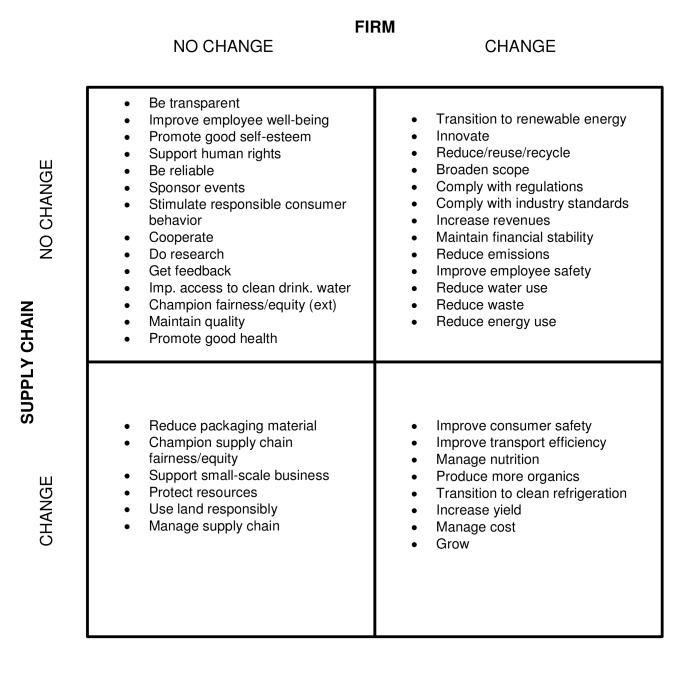
Core Behavioral Change in Firm and Supply Chain for Packaged Food Industry CSR Behavior.

To get a better idea of where the effort of the firms is going, we separate the codes based on the type of change that certain CSR practices require–that is, whether or not a firm has to alter their core production practices, or motivate others in the supply chain to do so. The results are shown in Fig. 2. Because we are judging the effort of specific behaviors, we include only the codes that are linked to such behaviors thereby omitting the eight aforementioned general codes.

Each of the four quadrants represents a combination of two assessments: Along the horizontal axis, we have a decision on whether or not the firm has to make a change in its own core behavior. Along the vertical access, we have the same decision for the supply chain.

In the **top left quadrant**, we find codes that require change in core behavior from neither the firm nor the supply chain. We also see that all of these codes can also be found in the “People” circle in Fig. 1. That is, activities that require no change in core firm behavior or core supply chain behavior tend to focus on people. Upon closer examination, it becomes clear that these activities are either community based, or basic expectations of internal or external stakeholders. Those that fall into the first category are relatively cheap. Sponsoring an event or educating farmers about environmentally friendly practices requires relatively little effort in terms of organization, employee hours, and money. Behaviors such as maintaining quality or doing research may require more significant and consistent input from the firm, but fall under the category of basic stakeholder expectations–that is, they would have to be done anyway to maintain stakeholder relationships (we explore this further in Section 5.3).

The codes in the **top right quadrant** are ones that *do* require changes in core firm behavior but do not require support from the rest of the supply chain. This does not mean that others in the supply chain cannot take on similar projects with the same CSR goals, but they do so independently of the packaged food firm. Notable is that most almost all codes relate to either planet or profit. Only the code “Improving employee safety” is about people. The motives behind these behaviors are certainly up for debate. All of these firms are certified by the International Organization for Standardization (ISO), which regulates some of these behaviors. This makes those behaviors normal business practice, a concept that is further explored in Section 5.3.

The **bottom left quadrant** represents behaviors that do not require any change in core firm behavior, but do require that the firm select supply chain partners with specific characteristics or that their current supply chain partners make changes in *their* core behavior. While the firm itself is not making core behavioral changes, it is possible that in some cases, the firm is encouraging or even pressuring their supply chain partners to behave a certain way. For the most part, this is unclear in the sustainability reports. General statements, such as the one below from a major beverage company, are typical among these documents.

*“We also work with suppliers to seek to reduce the carbon emissions and waste associated with our packaging.”*

We do not know how they “work with” suppliers or what the relationship is between the two firms, but we do know that there is some sort of inter-organizational agreement to make CSR-based changes. For the firm, such cooperation on virtually any CSR-related topic can be valuable in terms of public relations, while requiring no actual change from the firm itself. The behaviors are distributed over all over the three triple bottom line categories–there is no apparent pattern.

The **bottom right quadrant** includes only codes that require firm *and* supply chain cooperation. This means that a minimum of two organizations have to cooperate to make the change happen. As such, these changes require arguably the most effort to achieve. This is why we take a closer look at each of these behaviors in this section.

The first of them is to *Improve consumer safety*. In a supply chain as long as that of packaged food, this is only achievable through supply chain cooperation. Safety considerations can include the agriculture sector, storage, food laboratories, packaging companies, storage facilities, the firm itself, and virtually any other supply chain partner. *Manage nutrition* is a code that requires supply chain-wide effort for similar reasons. Nutrition is affected at various stages, including but not limited to agriculture, additive-producing laboratories, food processing, and storage. The next code on the list is *Produce more organics*, which means that every ingredient added to the product must be organic. It is obvious what that means for ingredients coming directly from the agriculture sector, but it also means that any additives derived from plants must come from organic plants. This can be complicated to control for products in the packaged food industry as it is common for these products to have twenty or more ingredients. *Improve transport efficiency* is deceiving because the name implies only a change in transport–a supply chain partner. But in reality, reducing transport distance can require drastic measures throughout the supply chain, including optimizing the location of production sites, vendor production sites, and distributors. This can mean anything from changing vendors to negotiating with new distributors, to building new factories. *Transition to clean refrigeration* requires more than just switching out a few refrigerators. In this industry, refrigeration is used in factories, warehouses, distribution points, and transport vehicles. Not only must some final products be refrigerated, but raw ingredients can require refrigeration as well. This means that cold chain must be consistently maintained for some items as they move between factories, warehouses, and retail points. Changing this equipment for more eco-friendly equipment is cost and labor intensive throughout the supply chain, and procedural or product changes may be required to reduce the need for refrigeration or adapt to the new refrigeration equipment. The final three codes are at least partially profit-focused, but cannot be accomplished without the involvement of the supply chain. To *Increase yield* means more output for less input. This can mean more efficient production processes but also a change to low-waste raw ingredients. To *manage cost* means decreasing costs throughout the supply chain so that the product can be brought to the consumer for a lower price. Beyond reducing production costs, the firm must also reduce input costs, transport costs, packaging costs, and warehousing costs (among others). The final code is to *grow*. The growth of a firm in the packaged food industry would also require others in the supply chain to grow in order to keep up with the needs of the firm. Again, the behaviors are distributed over all triple line categories; there is no apparent pattern.

From this analysis we can see behaviors that are solely about profit or planet require more effort from either the firm or the supply chain than a large number of people oriented behaviors. This is a possible explanation of why the people category is so elaborated upon: these behaviors are “low hanging fruit”–achieved with relative ease. Within the boundaries of the firm, most CSR challenges are about planet and profit. However, when supply chain partners get involved, effort is distributed all over the triple bottom line framework.

### 3. Normal Business Practice

**Fig 3:**
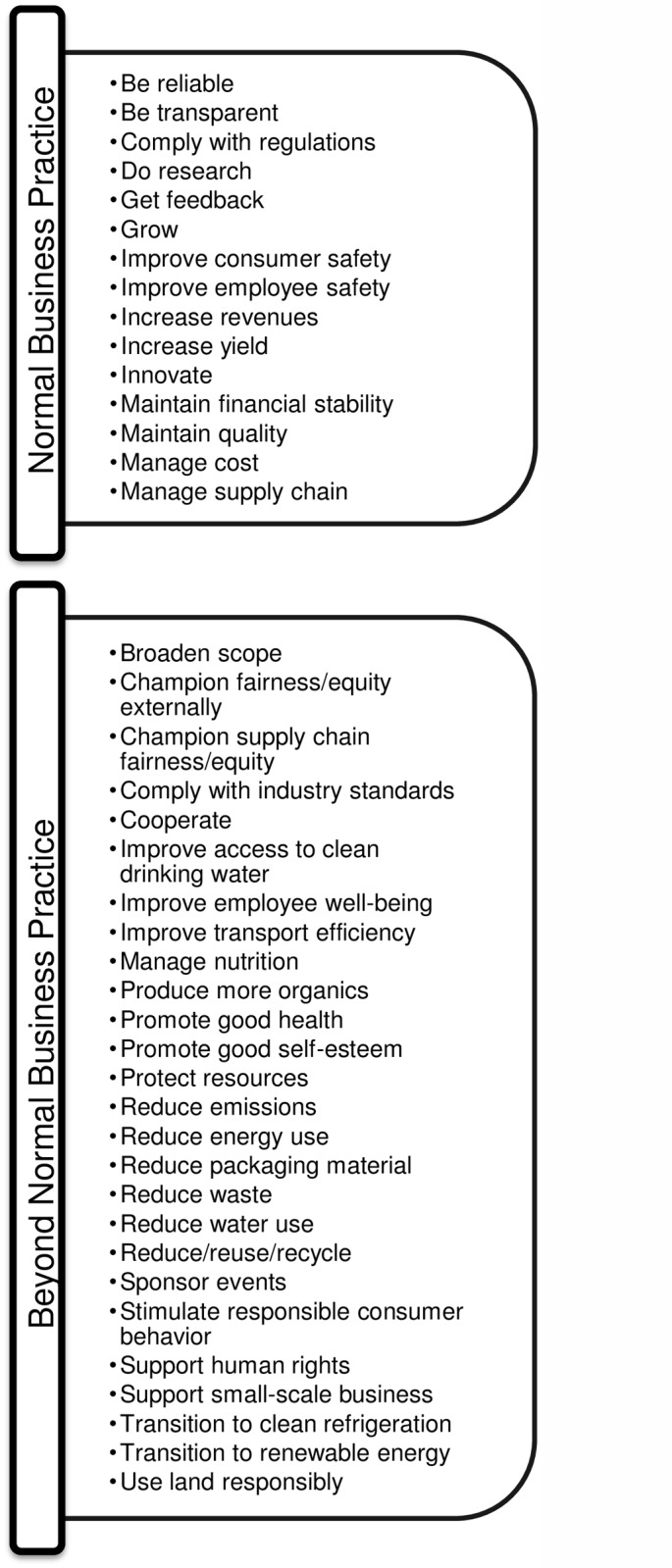
Normal Business Practice for Packaged Food Industry CSR Behavior.

All of the codes in the rightmost column and bottom row (Fig. 2) require either a significant level of effort on the part of the firm or a substantial level of cooperation throughout the supply chain. However, this does not necessarily mean that those efforts were initiated for the sole purpose of being responsible. It is possible that the firm would have implemented those behaviors anyway, either to comply with regulations or as part of the normal business practices that allow it to survive in the short-term. This is shown in Fig. 3.

The **first column** (Fig. 3) is titled “Normal business practice,” and represents behaviors that the firm would have likely engaged in regardless of the extent of its propensity for CSR, but chose to include in its sustainability reports anyway. Codes are placed here using two criteria. The first is that behaviors are to some extent mandated, and as such, the firm could be subject to loss of permits or excessive fines for non-compliance. Also in this column are codes for behaviors that are necessary for the firm to stay in business in the short-term. There is some subjectivity involved here. A code like “Get feedback” could probably find an arguable place in either column. A large corporation with many products may be fine without it in the short-term if it makes some lucky decisions and predictions about enough of its products. But we also imagine a scenario where business-destructing problems are missed due to lack of feedback from employees, consumers, or other stakeholders. Since the practical solution is to count on communication over luck, we call this behavior normal business practice.

We recognize that this category is probably underrepresented. Behaviors that are required for the long-term survival of the firm, for example, are not included. As such, there are behaviors that might not necessarily be “voluntary extras” that are not included in this column. Despite this, we can say with confidence that *at least* the behaviors in this column are normal business practice, and perhaps others as well.

The **second column** (Fig. 3), titled “Beyond normal business practice,” contains codes linked to behaviors that the firm may not have undertaken if not for an effort to be more responsible. In this category, we have placed all of the behaviors for which we could not make a “normal business practice” argument. We thereby conclude that any behavior, which is not normal business practice, goes beyond normal business practice.

This analysis shows that taking planet into consideration is not (yet) a requirement for firm survival and that these type of actions depend on the firm’s own intrinsic motivation or the need to manage stakeholders. An optimistic view about this is that corporate leaders in the industry do feel responsible for the environment and are willing to go the extra mile. Some interviewees claim that this is indeed the case. One respondent from a prominent condiment-focused firm states, in reference to sustainability,

*“I think from a company perspective, we always say it's part of the genes of the company”*

However, an interpretation that is more in line with stakeholder management is that firms display these behaviors because stakeholders expect it. By reporting these behaviors explicitly in their CSR reports, firms are responding to societal demand for more sustainable food products. One respondent from a prominent Europe-based firm says,

*“…there is much more of an awareness of the environment. In terms of consumers, that's also where their heads are going. Consumers are much more aware of the impact that people are having on the environment and how things are changing quite rapidly.”*

This implies that consumer demands are changing, forcing the industry to change as well.

## Discussion and Concluding Remarks

In this paper, we classified the self-identified CSR behaviors of firms in the packaged food industry according to three different schemes: (1) the triple bottom line approach, (2) the presence of change in core firm behavior of the firm itself or of others in the supply chain, and (3) whether the behavior qualifies as being outside of the firm’s normal business practice or is something that they might have done anyway. The results of this study demonstrate how the visible CSR efforts of a firm can be misleading at first glance.

In our first analysis, using the triple bottom line framework, we got a clear result. Even when considering the interdependencies of codes–that is, codes that were subject to more than one category–the “People” category was still markedly more full, leading to the preliminary conclusion that the CSR focus of firms in the packaged food industry is people (as opposed to planet or profit). In our second analysis, we found that the codes focusing on people require the least amount of real structural change, and as such, arguably the least amount of effort. The reason firms talk mostly about People-based behaviors is likely two-fold: they are easy to achieve, and to appease their stakeholders. These two explanations are different but complementary. It is possible that packaged food companies display specific, relatively easy CSR behaviors to strengthen the corporate image and to satisfy stakeholders. However, since it does not take much effort to conduct these behaviors, one can wonder if the firm can really distinguish itself in this fashion. Perhaps reporting extensively on such low-effort behaviors can come across as greenwashing–the overuse of sustainability-centered vocabulary for the purpose of marketing or public relations. Furthermore, reporting a lot of people behavior seems to have become more of an industry standard with which firms need to comply–a function of anthropocentric bias in sustainability reporting [48].

We also found that within the boundaries of the firm, core behavioral change (effort) is required mostly for planet-focused behaviors, but when it comes to the supply chain, core behavioral change is required for behaviors in all three categories without any noticeable trends. To explain this phenomenon, we resolve that systematizing behaviors within the boundaries of the organization is more manageable for People- and Profit-based behaviors than when organizational boundaries are crossed to reach others in the supply chain, at which point behaviors in all three categories become equally difficult to implement. As discussed in Section 2, packaged food industry supply chains can be exceedingly long, and managing or keeping up with the behavior of supply chain partners may become the primary obstacle for all categories of 3BL behaviors.

Finally, we found that most CSR behavior that is related to the planet is not an absolute requirement for survival and tends to fall into the “Beyond normal business practice” category. As such, we conclude that firms can better distinguish themselves from competitors by focusing more on Planet-based behavior. Based on our results, we see that packaged food companies have already identified quite a few opportunities for CSR. The People category is particularly well elaborated upon but a variety of other activities are also reported. Activities that are normal business practice, as per our definition, are a requirement for survival and are thus adopted by most or all firms in the industry. If a firm wants to stand out amongst its competitors as a responsible firm, it must adopt voluntary behaviors that are valued by society but not yet an industry standard that affects the survival of the firm–planet-based behaviors. However, if societal demand for planet-focused CSR persists, then the need to manage stakeholders will cause it to become normal business practice, next to people and profit. While this may be good for society, it will no longer be a distinguishing factor for the firm, resulting in the emergence of new CSR trends as firms attempt to distinguish themselves from competitors.

Adding the concepts of core firm behavioral change and normal business practice to 3BL can be a significant first step to more transparent sustainability reporting. By considering the effort and extra initiative required to execute specific CSR projects, stakeholders are better able to evaluate the CSR behavior of firms on the basis of their commitment to being more responsible rather than just the number of stakeholder-appeasing projects that they can name in their report. This study helps to provide qualitative criteria against which firms’ CSR behaviors can be measured and to create a more honest overview of CSR practice. Based on our findings, we have created a set of standardized tables, which firms can use to include these new attributes in their reporting practices. This form can be found in S2 Appendix. Additionally, this study provides packaged food companies insights into gaps and opportunities in their current CSR practice and reporting, and offers consumers and other stakeholders a better understanding of firms’ practices.

Though our study takes a big step towards better understanding CSR practices in the packaged food industry, there are still important questions that remain unanswered. Further research can look into substantiating the claims of CSR behavior. Furthermore, mapping CSR as we have done can serve as a base for studies on the motivations behind CSR practices. More insights are needed about the conditions under which firms will engage in CSR and the processes that lead to an industry wide diffusion of CSR practices. We also need to gain more insight into which types of CSR are most accepted by stakeholders, particularly consumers. Finally, as the industry develops its CSR practices to adopt the latest sustainable business models like Cradle to Cradle or Blue Economy, more research will be needed, using these frameworks as theoretical support. These research trajectories can help firms to understand the specific causes of their own behavior and can lead to policy strategies that manipulate the conditions under which the firms operate to encourage more responsible behavior [49].

Finally, we discuss some limitations of this study. First, there are some concerns when working with self-reported data, particularly sustainability reports. Because sustainability reports could serve a political or marketing-based agenda, it is difficult to assess how substantial these practices actually are. One can assume that the activities that are part of normal business practice are thoroughly carried out (presumably because they would have been so anyway,) but for the others, this is a difficult issue for which to control. To mitigate this effect, we augmented the data with interviews for triangulation. However, interviewees were only available for half of the firms in our study. Given the impact that this industry has on society, it is important that these behaviors are checked by an independent auditor. For consumers and stakeholders to be able to obtain accurate and valuable information from these reports, independent audit must become the new industry norm. With the Global Reporting Initiative and other third party certifying bodies, we are already seeing steps in this direction.

Another limitation is that much of our classification relies on our own interpretation of the codes. This is a common criticism in qualitative research. For this reason we have added a detailed explanation in S3 Appendix for why each code is categorized as it is. Moreover, we based our arguments as much as possible on the analyzed text, organizational theory, and tertiary information about the packed food industry. The final limitation is the focus on a single industry. While we maintain that this is both a valid and a necessary characteristic of our study (see Section 2), it does limit the generalizability of the research to other sectors. There are some sectors to which our results are more generalizable, namely sectors that are large, multinational, difficult to avoid, and highly impactful. This includes industries like energy and pharmaceuticals. Even so, the magnitude and global importance of the packaged food industry warrants a study of type.

## Supporting Information

S1 Appendix Interview Schedule. (DOCX)

S2 Appendix Reporting Tools. (DOCX)

S3 Appendix Coding Clarification. (DOCX)
