# Supplementary material for: Putting Your Money Where Your Mouth Is: Why Sustainability Reporting Based on the Triple Bottom Line Can Be Misleading
Source: PLoS One. 2015 Mar 20;10(3):e0119036. doi: 10.1371/journal.pone.0119036 (PMC4368568; doi:10.1371/journal.pone.0119036)
Supplement: S1 Appendix — (DOCX) [file pone.0119036.s001.docx]

## Appendix S1: Interview Schedule

The following questions are to be used as a guide for interviewing middle level managers within sustainability or CSR departments of packaged food firms. All topics must be addressed in as open-ended a manner as possible, and not necessarily in the order in which they are written.

1. What, to you, is “responsible behavior” in the food industry?
2. Who is responsible for consumer health?

Possible prompts:

Consumers

Government

Organized consumer groups

Corporations

Community organizations

Society as a whole

1. Should the government (further) intervene in the packaged food market? Why?
   1. If so, in what way?
2. Does your organization participate in CSR?
   1. In what way?

Possible prompts:

Health

Consumer Safety

Worker Safety

Environment

Water

Energy use

Recycling

Sustainability

- 1. Why?

Possible prompts:

Good/bad for business

Responsibility to stakeholders

Legal obligation

Corporate citizenship

Part of a community

Other firms are doing it

1. How does your CSR behavior affect your organization?

Possible prompts:

Sales

Climate/Culture

Response from

Employees

Shareholders

Customers

Suppliers

Peers

Others

1. What behaviors of your organization could be classified as “irresponsible”?
2. Does your organization reformulate products?
   1. For what reason/with what goal?
3. Tell me about innovation in your organization
   1. Resources
   2. Goals/reasons
   3. Consumer benefits vs. organization benefits
4. What responsibilities do corporations have to you as a consumer?
5. What responsibilities do corporations have to society?
6. Do corporations in the food industry have special responsibilities to the consumer (compared to corporations in other industries)?
   1. If so, what?
