# Supplementary material for: Putting Your Money Where Your Mouth Is: Why Sustainability Reporting Based on the Triple Bottom Line Can Be Misleading
Source: PLoS One. 2015 Mar 20;10(3):e0119036. doi: 10.1371/journal.pone.0119036 (PMC4368568; doi:10.1371/journal.pone.0119036)
Supplement: S2 Appendix — (DOCX) [file pone.0119036.s002.docx]

# Appendix S2: Reporting Tools

In this document are two tables that can be used by firms as a tool for more transparent sustainability reporting.

The *Overview Table* provides a visual summary of all of the firms’ CSR activities, and as such, their performance based on the attributes discussed in this paper. The *Initiative Description Table* gives firms an opportunity to defend or elaborate on the claims that they make for each individual attribute.

**Overview Table**

| Initiative NAME | Affects PEOPLE | Affects the PLANET | Affects PROFIT | Requires  CORE FIRM BEHAVIORAL CHANGE  (firm) | Requires  CORE FIRM BEHAVIORAL CHANGE (supply chain) | Goes beyond NORMAL BUSINESS PRACTICE | Please use this column for comments and clarification |
| --- | --- | --- | --- | --- | --- | --- | --- |
| Example initiative | X |  | X | X |  |  | *Here, we mention any remarkable characteristics or occurrences related to this initiative, and clarify our choices on the left side of the row.* |
|  |  |  |  |  |  |  |  |
|  |  |  |  |  |  |  |  |
|  |  |  |  |  |  |  |  |
|  |  |  |  |  |  |  |  |

**Initiative Description Table**

| NAME of initiative |  | | |
| --- | --- | --- | --- |
| Does this initiative affect PEOPLE, for example your stakeholders or society as a whole? Please consider both positive and negative implications. | ⧠ YES  ⧠ NO | Please explain or defend your claim. |  |
| Does this initiative affect PLANET, for example land, water, air, or natural resources? Please consider both positive and negative implications. | ⧠ YES  ⧠ NO | Please explain or defend your claim. |  |
| Does this initiative affect PROFIT, including both short- and long-term financial benefits? Please consider both positive and negative implications. | ⧠ YES  ⧠ NO | Please explain or defend your claim. |  |
| Does this initiative require a change in CORE FIRM BEHAVIOR? Please consider whether or not it requires changes to the production process or the way that your firm or supply chain partners do business. If so, please check “YES.” | **Firm**  ⧠ YES  ⧠ NO  **Supply Chain**  ⧠ YES  ⧠ NO | Please explain or defend your claim. |  |
| Is this initiative NORMAL BUSINESS PRACTICE? Please consider whether or not it is necessary for the short-term survival of your firm. If so, please check “YES.” | ⧠ YES  ⧠ NO | Please explain or defend your claim. |  |
