# Supplementary material for: Putting Your Money Where Your Mouth Is: Why Sustainability Reporting Based on the Triple Bottom Line Can Be Misleading
Source: PLoS One. 2015 Mar 20;10(3):e0119036. doi: 10.1371/journal.pone.0119036 (PMC4368568; doi:10.1371/journal.pone.0119036)
Supplement: S3 Appendix — (DOCX) [file pone.0119036.s003.docx]

# Appendix S3: Coding Clarification

The table below contains qualitative variables for the placement of each code, and a justification for each placement decision. The first six columns are dummy codes, where 0=no and 1=yes. The seventh column, contains a justification for the placement of that code within our three different frameworks.

PEOP is whether or not a code belongs to the “People” category of the triple bottom line.

PLAN is whether or not a code belongs to the “Planet” category of the triple bottom line.

PROF is whether or not a code belongs to the “Profit” category of the triple bottom line.

ΔFRM is whether or not a code requires a change in core firm behavior to accomplish.

ΔCHN is whether or not a code requires a change in the core behavior of one or more supply chain partners to accomplish.

NBP is whether or not a code is normal business practice.

In the “Remarks” column, abbreviations for our three frameworks are used as a means of “tagging” each justification.

G stands for “General” and is used to identify codes that are not linked to a specific CSR behavior

PPP is the triple bottom line framework and stands for “People, Planet, Profit”

FBC stands for “Firm Behavioral Change” and refers to the effort framework that assesses core firm behavioral change

NBP stands for “Normal Business Practice” and refers to the framework that categorizes codes based on whether or not they are normal business practice

|  | PEOP | PLAN | PROF | ΔFRM | ΔCHN | NBP | Remarks |
| --- | --- | --- | --- | --- | --- | --- | --- |
| Be a good corporate citizen | 1 | 0 | 1 | G | G | G | **PPP:** deals with the integration of the business into the community – people and profit. |
| Be more efficient | 0 | 1 | 1 | G | G | G | **PPP:** producing a higher output with lower input uses fewer natural resources and saves money – planet and profit. |
| Be reliable | 1 | 0 | 0 | 0 | 0 | 0 | **PPP:** about keeping consumers happy through consistency in the product. **FBC:** does not require core firm behavioral change (in fact, quite the opposite since the goal is sameness)  **NBP:** is normal business practice because it is necessary for short-term survival – when consumers buy a product, they trust that it will taste the same every time. This must remain the case for the firm to stay in business. |
| Be sustainable | 1 | 1 | 1 | G | G | G | **PPP:** the definition of sustainability is broad and can include the longevity of society, the planet, or the firm – people, planet and profit. |
| Be transparent | 1 | 0 | 0 | 0 | 0 | 0 | **PPP:** about keeping stakeholders (people) in the loop.  **FBC:** requires additional activity (information dissemination/availability) rather than a change in core firm behavior.  **NBP:** is normal business practice because it is, to a large extent, regulated. |
| Broaden scope | 0 | 0 | 1 | 1 | 0 | 1 | **PPP:** a broader range of products offers a larger target demographic and could lead to a potentially greater market share for the firm – profit.  **FBC:** new or changing products, by definition, require core firm behavioral change but do not necessarily involve the supply chain.  **NBP:** is not normal business practice – broadening scope may or may not be necessary for firm survival in the long term, but given a presently successful product line, it may not be immediately pressing. |
| Champion fairness/equity externally | 1 | 0 | 0 | 0 | 0 | 1 | **PPP:** specific to the fairness/equity of people.  **FBC:** deals with projects outside of its own firm and supply chain (external) so does not require any change in core firm behavior.  **NBP:** charity projects that do not affect the survival of the firm in the short-term - not normal business practice. |
| Champion supply chain fairness/equity | 1 | 0 | 0 | 0 | 1 | 1 | **PPP:** specific to the fairness/equity of people.  **FBC:** focus on the supply chain – includes behaviors like sourcing sustainably which could involve a change in the core behavior of the firm’s suppliers, but not so much in the core behavior of the firm itself.  **NBP:** stakeholder interest – may affect the long-term financial health of the firm, but not in the short-term – it is thus not normal business practice. |
| Comply with industry standards | 1 | 1 | 1 | 1 | 0 | 1 | **PPP:** depending on the industry standard in question, people, planet, and/or profit could all be affected  **FBC:** often (but not always) requires a change in the core behavior of the firm but supply chain partners may be subject to separate industry standards within their own industries.  **NBP:** compliance is not mandatory. In the long-term, non-compliance can affect a firm’s position among its peers, but there is no risk to firm survival in the short-term, which makes it not normal business practice |
| Comply with regulations | 1 | 1 | 1 | 1 | 0 | 0 | **PPP:** depending on the regulation in question, people, planet, and/or profit could all be affected.  **FBC:** often (but not always) requires a change in the core behavior of a firm. Supply chain partners, however, are often subject to separate regulations for their own respective industries.  **NBP:** This is considered normal business practice because non-compliance with mandatory standards could lead to the shutting down of a firm. |
| Cooperate | 1 | 1 | 1 | 0 | 0 | 1 | **PPP:** could regard people, planet, and/or profit.  **FBC:** in the context of the sustainability reports, this term is used generally and superficially. It is also an “on the side” activity, and as such, does not require a change in core behavior for the firm or supply chain partners.  **NBP:** goes beyond normal business practice, as operating uncooperatively would not necessarily doom a firm in the short-term. |
| Do no harm | 1 | 1 | 1 | G | G | G | **PPP:** highly general concept that could easily be based on people, planet, and/or profit. |
| Do research | 1 | 1 | 1 | 0 | 0 | 0 | **PPP:** depending on the subject of the research, could be based on people, planet, and/or profit.  **FBC:** an auxiliary activity to core business and as such, does not require a change.  **NBP:** in the packaged food industry, new products are (demonstrably) constantly appearing and competing with the old. While not releasing new products in the short-term may not doom the firm, not doing research will. Research can take years to yield a product and is required in the short-term for the development of new products in the long-term. |
| Educate | 1 | 0 | 0 | G | G | G | **PPP:** educating people affects people. |
| Get feedback | 1 | 1 | 1 | 0 | 0 | 0 | **PPP:** depending on the subject of the feedback can cover people, planet, and/or profit.  **FBC:** is done in parallel to the core behavior of the firm.  **NBP:** is essential in both the short- and long-term for firm survival as it is necessary to pick up major debilitating flaws in the behavior of the firm, making it normal business practice. |
| Grow | 0 | 0 | 1 | 1 | 1 | 0 | **PPP:** increasing scale leads to higher revenues - profit.  **FBC:** the firm must adapt to more product lines, increased demand, and reorganization, which requires a change in core firm behavior. Also, supply chain partners must adapt to the additional demand or workload, leading to a change in core firm behavior from supply chain partner(s).  **NBP:** in the case of a publicly traded company (such as those in our study), growth is an important factor in remaining competitive on the market keeping investors satisfied. |
| Improve access to clean drinking water | 1 | 0 | 0 | 0 | 0 | 1 | **PPP:** action targeted at underprivileged people.  **FBC**: does not affect the business – done in parallel. As such, requires no core behavioral change from the firm or supply change partners.  **NBP:** in no way affects the business of the firm or its prospects for survival – goes beyond normal business practice. |
| Improve consumer safety | 1 | 0 | 1 | 1 | 1 | 0 | **PPP:** benefits consumers (people) while simultaneously avoiding debilitating public relations (profit), as is demonstrably (EU horsemeat scandal, various US Salmonella outbreaks) the case with food-borne disease outbreaks and other comparable safety-related events.  **FBC:** diseases and safety issues can be introduced at any point in the supply chain, so maintaining safety requires manipulation of core firm behavior from the firm and supply chain partners.  **NBP:** safety issues could lead to the rapid demise of a firm – managing them is normal business practice, |
| Improve employee safety | 1 | 0 | 0 | 1 | 0 | 0 | **PPP:** benefits employees (people).  **FBC:** can require production processes (core firm behavior) to change in order to meet emerging safety regulations.  **NBP:** regulated to a large extent in all types of manufacturing, non-compliance with which could lead to the firm being shut down. |
| Improve employee well-being | 1 | 0 | 0 | 0 | 0 | 1 | **PPP:** benefits employees (people).  **FBC:** employee wellness programs are “side projects” that do not affect business and require no core firm behavioral change.  **NBP:** popular for competitiveness in the labor market but these projects do not dictate a firm’s short-term survival, so are not normal business practice. |
| Improve society | 1 | 0 | 0 | G | G | G | **PPP**: society is, by definition, comprised of people. |
| Improve transport efficiency | 0 | 1 | 1 | 1 | 1 | 1 | **PPP:** can save on fuel costs, affecting profit, while simultaneously reducing emissions – planet.  **FBC:** can require a change in *where* products are processed or where they are sold – core firm behavioral change. Can also require core behavioral change from supply chain partners dealing with transport, to improve the efficiency of their transport vehicles or processes.  **NBP:** still largely voluntary and has no direct affect on short-term survival. |
| Increase revenues | 0 | 0 | 1 | 1 | 0 | 0 | **PPP:** by definition or ‘revenue’, affects profit.  **FBC:** could mean reducing processing costs or increasing the value of products, which require firm behavioral change.  **NBP:** normal business practice as not doing so affects the immediate financial health and survivability of the firm. |
| Increase yield | 0 | 0 | 1 | 1 | 1 | 0 | **PPP:** means producing more for less, which reduces cost and adds profit.  **FBC:** can mean change in production process (core behavioral change of firm) or change in input (core behavioral change of supply chain partners).  **NBP:** directly affects bottom line, and contributes to competitiveness of the firm, making it necessary for survival and normal business practice. |
| Influence consumer behavior | 1 | 0 | 0 | 0 | 0 | 1 | **PPP:** the consumers being influenced are people.  **FBC:** done via marketing campaigns, which are parallel to core business and require no core behavioral change.  **NBP:** this relates to influencing consumers to behave more responsibly, not to buy the firm’s product. This has nothing to do with the short-term success for the firm and is not normal business practice. |
| Innovate | 1 | 1 | 1 | 1 | 0 | 0 | **PPP:** depends on the subject and type of innovation – can affect people, planet, and/or profit.  **FBC:** requires significant investment as well as changes or additions to products or processes (core firm behavioral change).  **NBP:** products in the food industry are rapidly evolving with fad diets and new information. As such, constant innovation is required for survival. |
| Maintain financial stability | 0 | 0 | 1 | 1 | 0 | 0 | **PPP:** financial health means bringing in sustainable profits.  **FBC:** as markets change, firms may need to change their strategies, including those related to products or processes – core firm behavioral change.  **NBP:** a financially unstable firm cannot survive, so firms must always strive to maintain financial stability. As such, this is normal business practice. |
| Maintain quality | 1 | 0 | 1 | 0 | 0 | 0 | **PPP:** the quality of products affect people and quality scandals affect profit directly (product recalls) and indirectly (maimed brand loyalty).  **FBC:** *improving* quality may require core firm behavioral change, but simply maintaining it requires none.  **NBP:** not maintaining quality leads to, in the best case, consumer distrust; in the worst case, illness and death. It is normal business practice, as not doing it would lead to the firm’s demise. |
| Manage cost | 1 | 0 | 1 | 1 | 1 | 0 | **PPP:** keeping product costs down is good for consumers and keeping production costs down is good for the profit of the firm.  **FBC:** keeping production costs down could include using more efficient input (change in core behavior of supply chain partners) or changing production processes (change in core firm behavior).  **NBP:** crucial to the bottom line of the firm in the short-term – normal business practice. |
| Manage nutrition | 1 | 0 | 0 | 1 | 1 | 1 | **PPP:** affects the food that is eaten by consumers – people.  **FBC:** can include a change in input or production – change in core firm behavior and core behavior of supply chain partners.  **NBP:** some consumers value nutrition so it may have long-term bottom line effects, but in the short term, it is not crucial as there will also be consumers who do not care about nutrition. This is not normal business practice. |
| Manage stakeholders | 1 | 0 | 0 | G | G | G | **PPP:** stakeholders are, by definition, people. |
| Manage supply chain | 1 | 1 | 1 | 0 | 1 | 0 | **PPP:** supply chain can be managed to participate in people-, planet-, and/or profit-maximizing behaviors.  **FBC:** requires no change in core firm behavior since cooperation with supply chain partners does not affect product or process. Supply chain partners may be required to make core behavioral changes as a result of management on the part of the firm.  **NBP:** firms must manage the supply chain to achieve supply chain wide goals, but also to keep their own costs manageable – a necessity for short-term survival. |
| Plan for the future | 1 | 1 | 1 | G | G | G | **PPP:** can affect people, planet, or profit depending on the subject of the future plans. |
| Process food responsibly | 1 | 1 | 1 | G | G | G | **PPP:** responsibility is a non-specific term that can include, for example, the health of people, the effects on the environment (planet), or the resulting financial health of the firm (profit). |
| Produce more organics | 1 | 1 | 0 | 1 | 1 | 1 | **PPP:** organic foods affect the health of people as well as the waste and run-off that are re-deposited into the environment – planet.  **FBC:** requires a change in the production of new products, as well as a change in sourcing since a product cannot be organic unless all ingredients are organic. This means a change in core firm behavior and the core behavior of supply chain partners both.  **NBP:** neither regulated nor a requirement for survival as the firm may be targeting a market that is not interested in organics. |
| Promote good health | 1 | 0 | 0 | 0 | 0 | 1 | **PPP:** affects the health or health related knowledge of people.  **FBC:** any sort of promotion or marketing is an “on-the-side” activity and does not require core behavioral change.  **NBP:** not regulated and does not necessarily affect firm survival at all, particularly if the firm focuses on snack foods or confections. |
| Promote good self-esteem | 1 | 0 | 0 | 0 | 0 | 1 | **PPP:** affects the self-esteem or self-esteem related knowledge of people.  **FBC:** any sort of promotion or marketing is an “on-the-side” activity and does not require core behavioral change.  **NBP:** can indirectly benefit the firm by promoting the idea that being content with being overweight is good self-esteem, and as such, indirectly influencing the consumption of snack foods and confections. But this is neither a direct effect nor a necessary one for short-term firm survival. |
| Protect resources | 0 | 1 | 0 | 0 | 1 | 1 | **PPP:** refers specifically to natural resources – planet.  **FBC:** does not require a change in product or process, but could require a change in the way that raw materials are sourced, which could require a change in the core behavior of supply chain partners.  **NBP:** there are some regulations in place regarding protected resources but regulations are minimal, and as such, resource-protecting behaviors go beyond normal business practice. |
| Reduce emissions | 0 | 1 | 0 | 1 | 0 | 1 | **PPP:** refers to greenhouse gas emissions in the atmosphere, which affect climate, and as such, planet.  **FBC:** requires amended processes or changes in equipment – core firm behavioral change.  **NBP:** while there are some regulations in some geographic locations, they are relatively minor and emission reducing behavior is, for the most part, voluntary. |
| Reduce energy use | 0 | 1 | 1 | 1 | 0 | 1 | **PPP:** using less energy can reduce greenhouse gas emissions (planet) while also saving the firm energy costs (profit).  **FBC:** requires amended processes or changes in equipment – core firm behavioral change.  **NBP:** not subject to regulations and while using less energy saves money, switching to more energy efficient equipment costs money. As such, this behavior may actually cost the firm money in the short-term. |
| Reduce packaging material | 0 | 1 | 1 | 0 | 1 | 1 | **PPP:** less packaging usually leads to less non-biodegradable waste (planet) while saving the firm money on packaging material (profit).  **FBC:** the firm producing the food may not have to make any changes beyond negotiations with the packaging company, but the packager (a supply chain partner) will require core behavioral change.  **NBP:** unregulated, and cost savings may not be worth the effort for the firm to renegotiate contracts or pressure suppliers making it unnecessary for short-term survival – not normal business practice. |
| Reduce waste | 0 | 1 | 1 | 1 | 0 | 1 | **PPP:** reduces the need to store space-taking, chemical-leaching substances (planet), while reducing waste-removal costs for the firm (profit).  **FBC:** requires the need for a change in materials or processes – core firm behavioral change.  **NBP:** as long as the firm can afford waste disposal, not reducing waste cannot jeopardize it in the short-term. |
| Reduce water use | 0 | 1 | 1 | 1 | 0 | 1 | **PPP:** conserves a natural resource (planet) while saving on water costs (profit).  **FBC:** could require a change in processes – core firm behavioral change.  **NBP:** water is a relatively inexpensive resource and conserving it may save on some costs but would not make or break a firm in the short-term. |
| Reduce/reuse/  recycle | 0 | 1 | 1 | 1 | 0 | 1 | **PPP:** affects planet by reducing waste, and could affect profit by saving on the cost of new materials.  **FBC:** core behavioral change may be required due to a change in processes – particularly in the case of reuse and recycle.  **NBP:** this may or may not save the firm money and while policy exists in some places to encourage this behavior, it is not regulated. As such, it is not normal business practice. |
| Sponsor events | 1 | 0 | 1 | 0 | 0 | 1 | **PPP:** affects the people participating in the event while simultaneously marketing the firm (profit).  **FBC:**  no core firm behavioral change is required, as an event is, by definition, a “side project”.  **NBP:** does not affect the business of the firm, and costs the firm money with uncertain results. It may play a role in stakeholder confidence but is not required for short-term survival. |
| Support human rights | 1 | 0 | 0 | 0 | 0 | 1 | **PPP:** humans rights are directly about people.  **FBC:** done with side projects or donation to outside charities and NGOs – no core firm behavioral change required.  **NBP:** neither regulated nor necessary for short-term survival – not normal business practice. |
| Support small-scale business | 1 | 0 | 0 | 0 | 1 | 1 | **PPP:** provides a more stable living for the people who own small-scale businesses  **FBC:** requires no core firm behavioral change, but could require core behavioral change from small-scale businesses (supply chain partners) so that they can conform to the larger-scale needs of packaged food firms.  **NBP:** firms’ supply-chain relationships are not regulated and not supporting small-scale business is often not a short-term death sentence – it is not normal business practice. One consideration is that it *may* sometimes be the cheapest or only option for the firm, particularly in developing countries. |
| Transition to clean refrigeration | 1 | 1 | 0 | 1 | 1 | 1 | **PPP:** switching to less toxic refrigerants affects the health of people and the environment (planet) and switching to lower-energy refrigeration units could reduce emissions (planet).  **FBC:** refrigeration units exist throughout the supply chain to maintain cold chain. As such, this requires core firm behavioral change and core behavioral change from supply chain partners.  **NBP:** though this could be indirectly affected by regulations (regulations on emissions exist in some parts of the world), for the most part, this behavior is voluntary and does not affect short-term firm survival. |
| Transition to renewable energy | 0 | 1 | 0 | 1 | 0 | 1 | **PPP:** reduces emissions (planet).  **FBC:** requires significant structural and procedural change in manufacturing facilities – core firm behavioral change.  **NBP:** not yet regulated and difficult to accomplish if buying energy from a large-scale grid. As such, this is not normal business practice. |
| Use land responsibly | 0 | 1 | 0 | 0 | 1 | 1 | **PPP:** considers impacts of land use on environment – planet.  **FBC:** does not require core firm behavioral change but could require procedural change from farmers in the supply chain – core behavioral change of supply chain partners. |
